# Impact of Learning Style on Medical Students' Satisfaction With Hands-On Procedural Training

**DOI:** 10.7759/cureus.87543

**Published:** 2025-07-08

**Authors:** Hunter Cohn, Marley Sternberg, Madeline Evans, Swetha Reddy, Emily Vu, Katiya Barkho, Ward Hedges, Sabrina Khan, Daniel DeWeert

**Affiliations:** 1 Medical Education, Wayne State University School of Medicine, Detroit, USA

**Keywords:** customized medical education, innovation in medical education, multimodal learning styles, preclinical medical students, procedural skills training

## Abstract

Introduction

Hands-on procedural training is a cornerstone of medical education, yet little is known about how individual learning styles influence students' perceived relevance of procedural training. We explored the impact of learning styles on pre-clerkship medical students' perceptions of hands-on procedural training.

Materials and methods

A total of 151 pre-clerkship medical students (72% response rate; 66 males, 85 females) participated in an optional procedural training day comprising three one-hour workshops covering 22 different procedures. Participants completed an exit survey assessing their learning style per Kolb's Experiential Learning Styles (Accommodating, Assimilating, Converging, and Diverging) and satisfaction with the training, including engagement, enjoyment, and effect on their career outlook. One-way analysis of variance (ANOVA) and Tukey's honestly significant difference post hoc test were used to compare satisfaction levels of students based on learning style.

Results

The 151 survey respondents comprised 34 Accommodating (23%), 25 Assimilating (17%), 66 Converging (44%), and 26 Diverging learners (17%). Across all learning styles, students averaged high scores for satisfaction, with the mean exceeding 6 on a 7-point Likert scale for engagement, enjoyment, and future career outlook. Between learning groups, Assimilating and Accommodating learners reported the highest enjoyment scores, while Diverging learners reported lower enjoyment compared to these groups (p<0.05). No statistically significant differences were observed between learning styles for engagement or future career outlook. Students who attended sessions aligned with their preferred specialty reported higher satisfaction, particularly among Accommodating, Converging, and Diverging learners (p<0.01). While Kolb's Experiential Learning Model provided a framework for exploring learner variation, its limitations are recognized, and unmeasured factors such as prior procedural experience or baseline interest may have influenced satisfaction. The absolute differences between groups, however, were small, suggesting limited practical or educational significance despite statistical significance.

Conclusions

Hands-on procedural training was well-received by pre-clerkship students across all Kolb learning styles, with consistently high satisfaction scores. While some statistically significant differences in enjoyment emerged between learner groups, these differences were small and unlikely to warrant substantial modification of current instructional approaches. This study contributes to the limited literature on how learning styles influence early procedural medical education, suggesting that hands-on procedural training can serve diverse learning styles effectively without extensive adaptation. Future research may explore whether incorporating minor reflective elements could further optimize engagement for certain learner groups.

## Introduction

Hands-on procedural training is a cornerstone of medical education, equipping students with the technical skills and confidence essential for effective clinical practice [1]. Early exposure not only promotes technical proficiency but also offers valuable insight into medical specialties, helping inform students' career decisions [2,3]. For pre-clerkship students, these experiences establish a strong foundation for clinical rotations, where procedural skills are applied in real patient care settings [4-6]. Given the importance of procedural competence, optimizing training programs during students' pre-clerkship years is critical to maximize student engagement, skill acquisition, and long-term clinical proficiency [1].

Medical students exhibit diverse learning habits and preferences, suggesting the need for inclusive educational strategies that accommodate different learning styles [7]. Kolb's Experiential Learning Model offers one such framework, categorizing learners into four distinct styles: Accommodating, Assimilating, Converging, and Diverging [8]. These styles reflect differing approaches to processing information, from reflective observation to active experimentation. Kolb's model has been widely applied in nursing and medical education to characterize learner populations and inform preferences for teaching formats such as lectures, hands-on activities, and group discussions [9,10]. Understanding how learning styles influence responses to procedural training may inform the development of more effective, learner-centered instructional strategies [7].

Prior studies have examined learning styles in relation to academic performance and didactic learning. For example, Ogut et al. demonstrated associations between learning styles, study duration, and anatomy performance, while Newton et al. cautioned against the persistent endorsement of learning styles despite some empirical support [11,12]. Nevertheless, little is known about how learning styles affect responses to hands-on procedural education, which differs substantially from classroom-based learning. While it is known that Converging and Accommodating learners, who thrive in problem-solving environments, are overrepresented in medical education and often perform well academically [13-15], whether these tendencies translate to procedural satisfaction remains unclear. Moreover, learning styles are not fixed traits; they may evolve depending on instructional methods and the interplay between didactic and experiential learning [16,17]. This dynamic nature underscores the need to explore learning styles specifically within procedural training in preclinical education [11].

We therefore aimed to assess whether medical students' learning styles, as categorized by Kolb's framework, are associated with differences in engagement, enjoyment, and future career outlook following a one-day hands-on procedural training event. In doing so, we also sought to evaluate whether tailoring instruction to learning styles or considering other contextual factors is necessary to enhance satisfaction with procedural education.

## Materials and methods

Study design and participants

This was a cross-sectional survey study conducted at Wayne State University School of Medicine in Detroit, Michigan. The study focused on pre-clerkship medical students who participated in an optional, one-day procedural training event held outside of the required curriculum. The total pre-clerkship student body included 600 students (approximately 300 in each of the first- and second-year classes). Of these, 210 students attended at least one workshop during the event, resulting in a participation rate of 35% relative to the total pre-clerkship cohort.

Of the 210 event participants, 151 students (72% survey response rate) completed a post-event survey and were included in the analysis. Among survey respondents, 66 identified as male and 85 as female, with 80% representing first-year students and 20% representing second-year students. Participation in the event was voluntary, and students self-selected into both the training day and their specific workshop sessions.

Procedural workshops and specialty preference

Students were provided a list of 22 procedural workshops in advance and selected up to three sessions to attend. Workshops covered a range of procedures associated with multiple medical specialties, including suturing, central line placement, intubation, fracture stabilization, and dermatologic biopsies. Prior to the event, students indicated their current specialty of interest via an electronic pre-event form. This information was later used to explore whether attending workshops aligned with specialty preferences influenced satisfaction outcomes.

Data collection

Following the procedural training, participants completed an exit survey assessing both their learning preferences and their perceptions of the training sessions. Students were presented with brief, written descriptions of the four Kolb learning styles, namely, Accommodating, Assimilating, Converging, and Diverging, and asked to self-identify their dominant learning style category. The survey then included six items rated on a 7-point Likert scale (1=strongly disagree; 7=strongly agree), grouped into three satisfaction domains. More detail is provided below. 

Kolb's Learning Style Identification

**Figure 1:**
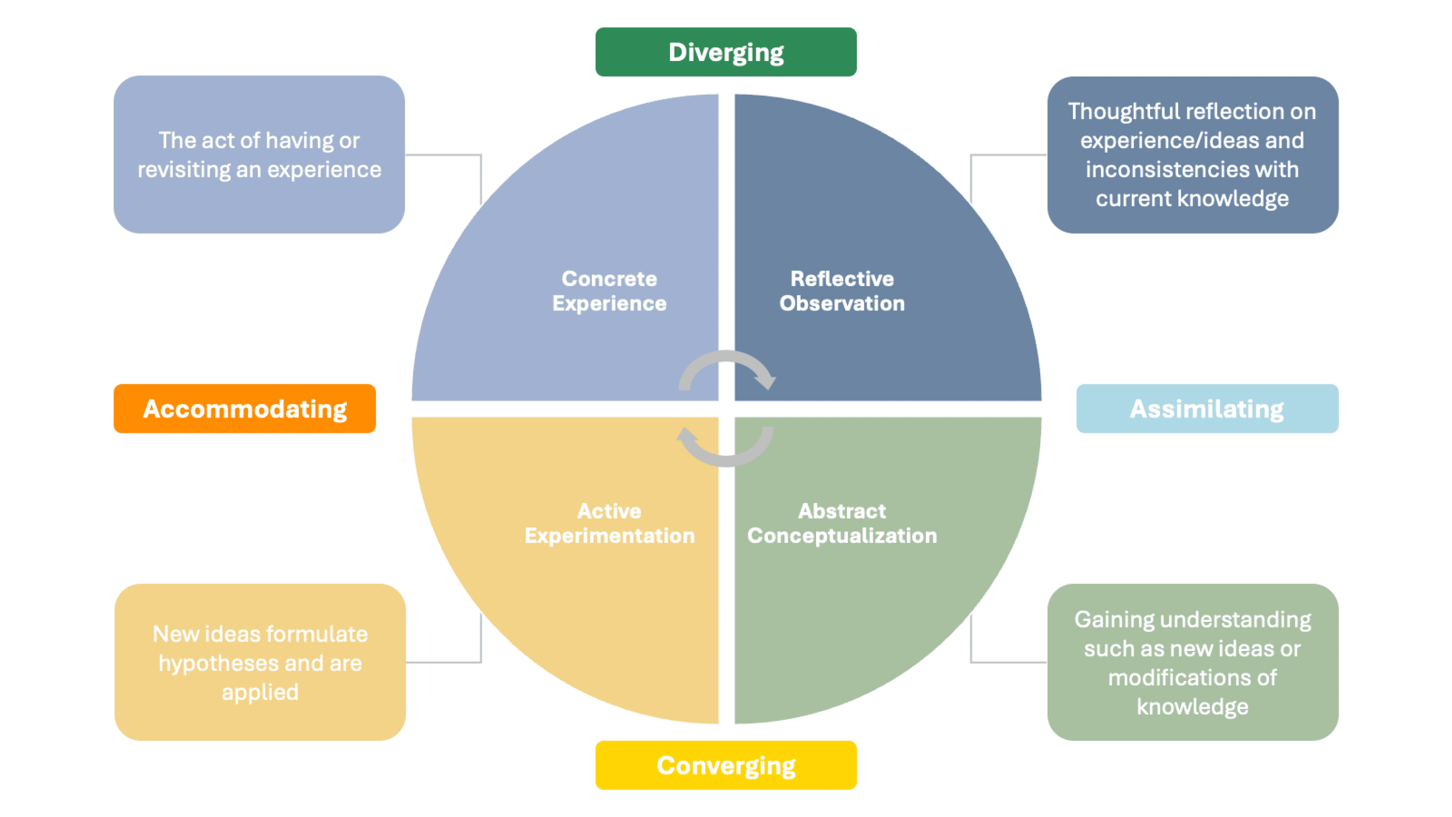
Experiential Learning Model of Kolb's learning styles

Students were provided the information included in Figure 1 and Table 1 and asked to self-identify their Kolb learning style (Diverging, Assimilating, Converging, or Accommodating). 

**Table 1:** Description of Kolb's learning styles provided to medical students participating in hands-on procedural learning sessions This table describes the four learning styles according to Kolb's Experiential Learning Theory: Accommodating (G1), Assimilating (G2), Converging (G3), and Diverging (G4).

Learning style	Learning style descriptions
Accommodating (G1)	Prefer being very hands-on and acting on instinct over logic. Rely on others for initial information and then test themselves.
Assimilating (G2)	Prefer synthesizing information into a logical and straightforward format. More about the facts than different perspectives. Value theory over action.
Converging (G3)	Prefer completing tasks and exhibiting practicality over interpersonal learning and are experiment-oriented.
Diverging (G4)	Prefer watching over action, looking at ideas from different perspectives, using imagination, and information gathering.

Session feedback was grouped according to the below categories. 

Engagement: "I was motivated to learn in today's session" and "I was engaged throughout the session".

Enjoyment: "I enjoyed this session" and "This session was a valuable use of time".

Future outlook: "Learning this procedure gave me a better understanding of this specialty" and "Today's session made me more knowledgeable about which specialties I want to pursue".

Data analysis

Survey responses were summarized using means and standard deviations. Composite scores were calculated for each satisfaction domain by averaging the two items within each category, generating summary measures for engagement, enjoyment, and future outlook. This approach was intended to reduce item-level variability and improve the stability of domain-level comparisons.

For primary analyses comparing satisfaction across learning styles, one-way analysis of variance (ANOVA) was used, followed by Tukey's honestly significant difference (HSD) post hoc tests for pairwise comparisons. For specialty alignment analyses, two-tailed independent-sample t-tests were used to compare satisfaction between students who attended sessions aligned with their stated specialty interest and those who did not.

Each survey item response was treated as an independent data point in aggregate analyses to allow domain-specific comparisons across multiple dimensions of satisfaction. This analytic choice allowed for greater granularity in exploring how individual components of satisfaction may vary across learning styles. However, this approach increased the number of observations relative to the number of unique participants and may have inflated statistical power, increasing the risk of type I error. We have explicitly acknowledged this limitation in the interpretation of findings.

To supplement p-value reporting and better contextualize the practical significance of observed differences, effect sizes were calculated for key comparisons. For ANOVA results, eta-squared (η²) was used to estimate the proportion of variance explained by learning style. For pairwise t-test comparisons (specialty alignment analyses), Cohen's d was calculated to quantify the magnitude of between-group differences. Effect size interpretation followed conventional thresholds: η² values <0.06 considered small and Cohen's d values ~0.2 small, ~0.5 medium, and ~0.8 large.

Ethics approval

The study was reviewed and deemed exempt from full Institutional Review Board (IRB) review due to its educational quality improvement focus (Wayne State University School of Medicine Institutional Review Board, Protocol ID: 2024 088). All procedures were conducted in accordance with institutional standards and the ethical principles of the 1964 Declaration of Helsinki and its later amendments. Informed consent was obtained from all participants prior to survey completion.

## Results

Participant characteristics

**Figure 2:**
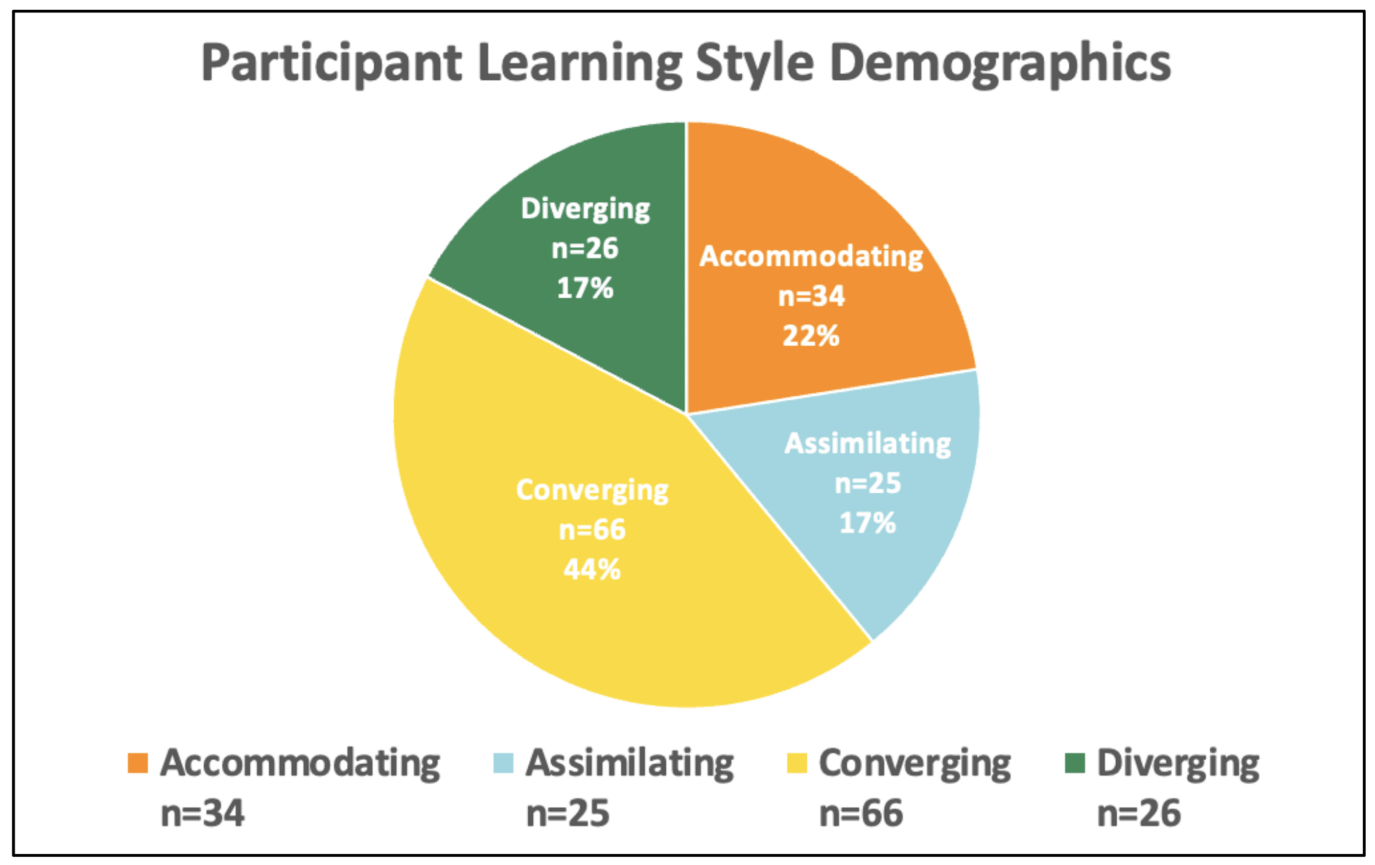
Learning styles of survey respondents according to Kolb's Experiential Learning Model

Of the 600 eligible pre-clerkship students, 210 participated in the optional procedural training event (35% participation rate), and 151 students completed the post-event survey (72% survey response rate). Among survey respondents, 66 (44%) identified as male and 85 (56%) as female; 80% were first-year students and 20% were second-year students. Regarding learning styles, 44% identified as Converging (n=66), 22% as Accommodating (n=34), 17% as Assimilating (n=25), and 17% as Diverging (n=26) (Figure 2).

Engagement with procedural training

Mean scores for the two engagement items were highest for Assimilating learners, with mean scores being 6.48±0.8 for question 1 and 6.44±1.0 for question 2. These were followed by Accommodating learners (6.42±0.9 and 6.31±1.1), Converging learners (6.30±1.1 and 6.33±1.1), and Diverging learners (6.28±1.1 and 6.23±1.2) (Table 2). No significant differences were observed between groups for engagement (Table 2). The corresponding effect size was small (η²=0.02).

**Table 2:** Post-training survey responses by students' self-reported learning styles This table represents survey responses assessing engagement, enjoyment, and future career outlook across sessions. Each survey prompt is stratified into a Kolb learning style. Survey responses ranged from 1 (strongly disagree) to 7 (strongly agree). _a_The highest score in each row is in italics. _b_Pairwise comparisons of all four learning styles were done per Tukey's honestly significant difference post hoc test. Only significant results are included to save space. nd: no significant differences observed; SD: standard deviation

Category	Survey item	Survey scores by learning style_a_ (mean±SD)				P-value_b_
		Accommodating (G1) (n=34)	Assimilating (G2) (n=25)	Converging (G3) (n=66)	Diverging (G4) (n=26)	
Engagement	I was motivated to learn in today's session.	6.42±0.9	6.48±0.8	6.30±1.1	6.28±1.1	nd
	I was engaged throughout the session.	6.31±1.1	6.44±1.0	6.33±1.1	6.23±1.2	nd
Enjoyment	I enjoyed this session.	6.45±0.9	6.47±0.9	6.32±1.1	6.03±1.2	G1 v G4 p<0.05
						G2 v G4 p<0.05
	This session was a valuable use of time.	6.42±1.0	6.41±1.2	6.26±1.2	6.11±1.3	nd
Future outlook	Learning this procedure gave me a better understanding of this specialty.	6.16±1.2	6.36±1.1	6.19±1.1	6.08±1.2	nd
	Today's session made me more knowledgeable about which specialties I want to pursue.	6.30±1.1	6.52±0.8	6.24±1.1	6.33±1.1	nd

Enjoyment of procedural training

Assimilating learners and Accommodating learners had the highest mean scores for the two enjoyment items. Assimilating learners had mean scores of 6.47±0.9 for the first question and 6.41±1.2 for the second question, followed by Accommodating learners at 6.45±0.9 and 6.42±1.0, Converging learners at 6.32±1.1 and 6.26±1.2, and Diverging learners at 6.03±1.2 and 6.11±1.3 (questions are available in "survey item" column in Table 2). Diverging learner scores were significantly lower than the Accommodating and Assimilating learner scores for the first enjoyment question (p<0.05) (Table 2). However, the effect size for this difference was small (Cohen's d=0.35), indicating limited practical significance.

Future outlook after procedural training: impact on career interests

Assimilating learners again reported the highest mean score across both questions that assessed future career outlook, with mean scores of 6.36±1.1 for the first question and 6.52±0.8 for the second question, followed by Accommodating learners at 6.16±1.2 and 6.30±1.1, Converging learners at 6.19±1.1 and 6.24±1.1, and Diverging learners at 6.08±1.2 and 6.33±1.1. No significant differences were observed between groups for either future career outlook item (Table 2). Again, the effect size was small (η²=0.01). 

Medical specialty session comparisons

Additionally, within each learner category, composite scores that aggregated and averaged the six questions' responses were compared by medical specialty procedure. Notably, Accommodating learners who attended the hematology/oncology or pediatrics sessions had significantly lower scores than those who attended other specialty sessions (both p<0.01). Diverging learners who attended psychiatry sessions had significantly lower scores than Diverging learners who attended other specialty training sessions (p<0.01). Students in the other two learning style groups did not have significantly different scores across specialty sessions (Table 3). Small to moderate effect sizes were observed with Cohen's d ranging from 0.30 to 0.45. 

**Table 3:** Composite survey responses by learning style and medical specialty This table represents the aggregate engagement, enjoyment, and future career outlook scores based on the specialty sessions which students attended. It is segmented by student learning style. _a_The highest scores in each row are in italics. _b_Pairwise comparisons of all four learning styles were done per Tukey's honestly significant difference post hoc test. Only significant results are included to save space. _c_Pairwise comparisons by learning style were conducted for students attending specific specialty sessions using Tukey's honestly significant difference post hoc test; only significant results are included to save space. Survey responses ranged from 1 to 7 with higher numbers indicating higher satisfaction. nd: no significant differences observed; ENT: otolaryngology; Hem/Onc: hematology/oncology; IV: intravenous; OB-GYN: obstetrics-gynecology; Pap smear: Papanicolaou test; Peds: pediatrics; SD: standard deviation

Self-reported medical specialty (procedural session attended)	Composite survey scores by learning style_a_ (mean±SD)				P-values_b_
	Accommodating (G1)	Assimilating (G2)	Converging (G3)	Diverging (G4)	
Cardiology (echocardiography)	6.23±0.9 (n=30)	6.67±0.5 (n=18)	6.08±1.3 (n=102)	5.77±1.4 (n=48)	G2 v G4 p<0.05
Cardiothoracic surgery (chest tube insertion, cardiac suturing)	6.61±0.6 (n=60)	7.0±0.0 (n=12)	6.73±0.4 (n=30)	5.77±1.4 (n=24)	G2 v G4 p<0.01
					G3 v G4 p<0.05
Dermatology (shave and punch biopsies)	6.37±0.9 (n=30)	6.79±0.4 (n=12)	6.86±0.5 (n=96)	6.86±0.5 (n=96)	G1 v G2 p<0.01
					G1 v G3 p<0.01
					G1 v G4 p<0.01
Emergency medicine (IV-guided ultrasonography, intubation)	6.75±0.5 (n=84)	6.67±0.5 (n=18)	6.70±0.7 (n=120)	6.57±0.8 (n=54)	nd
ENT (audiometers, ENT suturing)	6.14±1.2 (n=48)	6.04±1.3 (n=24)	5.42±1.5 (n=48)	6.16±0.7 (n=18)	nd
General surgery (classic suturing)	6.36±0.9 (n=36)	5.90±1.5 (n=30)	6.50±0.8 (n=72)	6.63±0.5 (n=36)	G2 v G3 p<0.05
					G2 v G4 p<0.05
_c_Hematology/oncology (brachytherapy)	4.50±2.6 (n=12)	6.00±1.7 (n=30)	6.30±0.9 (n=66)	6.31±0.9 (n=42)	G1 v G2 p<0.01
					G1 v G3 p<0.01
					G1 v G4 p<0.01
OB-GYN (Pap smear, contraceptive implant insertion, breast exam)	6.15±1.2 (n=48)	6.14±0.6 (n=30)	6.12±0.8 (n=90)	7.00±0.0 (n=12)	G1 v G4 p<0.05
					G2 v G4 p<0.05
					G3 v G4 p<0.05
Orthopedic surgery (fracture fixation, joint stabilization)	6.45±0.9 (n=60)	6.63±0.6 (n=36)	6.57±0.9 (n=84)	6.33±0.8 (n=24)	nd
_c_Pediatrics (newborn assessment, pediatric airway management)	5.39±1.5 (n=36)	6.47±0.8 (n=42)	6.01±1.3 (n=78)	5.33±1.3 (n=12)	G1 v G2 p<0.05
					G2 v G4 p<0.05
Plastic and reconstructive surgery (microsurgery)	6.15±1.1 (n=48)	6.45±0.9 (n=42)	5.99±1.2 (n=90)	5.83±1.3 (n=30)	nd
_c_Psychiatry (engaging patients experiencing behavioral escalation)	6.00±0.0 (n=6)	6.03±1.3 (n=30)	5.36±1.3 (n=90)	4.88±1.5 (n=24)	nd
Vascular surgery (central line access)	6.52±0.9 (n=42)	6.44±0.9 (n=18)	6.51±0.6 (n=102)	6.46±0.8 (n=54)	nd
P-values_c_	Hem/Onc p<0.01; Peds p<0.01	nd	nd	Psychiatry p<0.01	

Impact of attending a preferred medical specialty session

The study also examined whether attending a session that was aligned with students' stated specialty preference was associated with their reported satisfaction with the training. Students who attended sessions in their desired specialty reported slightly higher mean scores across all three satisfaction categories compared to those who attended non-desired specialty sessions. For example, Accommodating learners who attended desired specialty sessions reported a mean engagement score of 6.63±0.7 versus 6.25±1.1 for those who attended non-desired sessions (p<0.01). Similar results were also observed for Converging and Diverging learners (both p<0.01). Assimilating learners, however, did not have significantly different overall scores based on having attended sessions that aligned with their chosen specialty (p=0.22) (Table 4). However, effect sizes for these differences were small to moderate (Cohen's d ≤0.50), and findings should be interpreted with caution given the small subgroup sizes involved.

**Table 4:** Composite survey responses by learning style and attendance in procedural training matching desired medical specialty This table represents the aggregate engagement, enjoyment, and future career outlook scores grouped according to whether the student attended the specialty session which matched their desired specialty. It is segmented by student learning style. N numbers exceed the number of participants because each participant noted only one desired specialty but attended three different procedural sessions. _a_The highest scores in each row are in italics. _b_P-values are pairwise comparisons of all four learning styles done per Tukey's honestly significant difference post hoc test. Only significant results are included to save space. _c_P-values from two-tailed t-test comparing Yes versus No groups for each individual learning style. Survey responses ranged from 1 to 7 with higher numbers indicating higher satisfaction. nd: no significant difference observed; SD: standard deviation

Participated in desired specialty procedural session?	Composite survey scores by learning style_a_ (mean±SD)				P-values_b_
	Accommodating (G1)	Assimilating (G2)	Converging (G3)	Diverging (G4)	
Yes: matched to desired specialty	6.63±0.7 (n=150)	6.36±1.2 (n=138)	6.52±0.8 (n=246)	6.54±0.9 (n=96)	nd
No: not matched to desired specialty	6.25±1.1 (n=312)	6.48±0.8 (n=936)	6.20±1.1 (n=378)	6.08±1.2 (n=462)	G1 v G2 p<0.05
					G2 v G3 p<0.01
					G2 v G4 p<0.01
P-value_c_	p<0.01	p=0.22	p<0.01	p<0.01	

## Discussion

In this survey study, we examined how Kolb's learning styles (Accommodating, Assimilating, Converging, and Diverging) influence pre-clerkship medical students' perceptions of hands-on procedural training, a key component of clinical education that differs meaningfully from didactic or anatomy laboratory instruction. While learning styles have been extensively studied in the context of academic performance, their role in early experiential procedural training remains underexplored [18-20].

Across all learning styles, students reported high satisfaction, engagement, and enjoyment, suggesting that hands-on procedural training is broadly effective and well-received. Although statistically significant differences emerged, particularly with Diverging learners reporting slightly lower enjoyment than Accommodating and Assimilating learners, calculated effect sizes were small. This indicates that while differences are measurable, their educational or clinical relevance may be limited. Collectively, these findings suggest that the current instructional format is broadly inclusive, with modest opportunities for targeted refinements that could enhance engagement for select learner groups.

While Kolb's model provides a useful framework for examining individual learning preferences, its psychometric validity remains debated within medical education. Critics have noted inconsistent empirical support for learning style frameworks broadly, raising concerns about rigid application in instructional design [7,10,11,13-15,17,19]. Our findings contribute to this ongoing dialogue by demonstrating that even in a highly experiential domain that is theoretically suited for learning style differentiation, satisfaction differences were minor. High-quality procedural instruction appears capable of supporting diverse learner profiles without necessitating extensive tailoring to individual learning styles.

Engagement, enjoyment, and learning styles

Our findings demonstrate that procedural training was well-received across all Kolb's learning styles. Assimilating learners, who prefer structured, information-rich environments, reported the highest engagement, reflecting their affinity for clear frameworks and organized instruction. In contrast, Diverging learners, who favor reflective observation, reported slightly lower engagement, likely reflecting the emphasis on active experimentation within these workshops.

These findings are consistent with prior research showing that aligning content delivery with individual learning modalities, that is, visual, auditory, or kinesthetic, can influence student engagement [21-23]. Procedural training naturally aligns with kinesthetic learning, but our results suggest that adding components tailored to reflective learners may further optimize the learning environment, particularly for Diverging learners [24].

This pattern aligns with Kolb's Experiential Learning Cycle, which emphasizes concrete experience, reflective observation, abstract conceptualization, and active experimentation [22]. While the workshops focused heavily on active experimentation, Diverging learners' preference for observation and reflection may have been underrepresented. Simple refinements, such as brief reflective debriefs, pre-session demonstration videos, or mid-session peer check-ins, could help integrate the reflective observation phase into procedural sessions without altering their core structure.

While overall satisfaction scores were high and observed differences between groups were small, these targeted adjustments represent opportunities for incremental improvement, fine-tuning rather than overhauling a format that is already broadly effective across diverse learner profiles.

Future outlook and career interests

Our study also evaluated the influence of procedural training on students' confidence in evaluating their fit within different specialties. Across all learning styles, participants reported increased confidence following the sessions, highlighting the potential role of early experiential exposure in shaping career exploration.

This observation complements prior work by Bitran et al., who found that Converging learners often gravitate toward surgery and primary care, while Assimilating learners are more likely to pursue internal medicine, pediatrics, and psychiatry [25]. While our study did not track actual specialty choice, it provides preliminary evidence that early procedural experiences can positively shape specialty interests prior to clerkship, potentially reinforcing learning style tendencies identified in later stages of training.

Specialty preferences and learning styles

Students were generally receptive to all procedural workshops, regardless of their stated specialty interests. However, when sessions aligned with a student's preferred specialty, Diverging, Converging, and Accommodating learners reported significantly higher satisfaction, though overall scores remained high, averaging above 6 out of 7 across all groups. This suggests that while specialty alignment modestly enhances satisfaction, the quality of instructional design remains the dominant driver of positive learner experience.

These findings further echo Bitran et al.'s work linking learning style with specialty orientation [25] and suggest that even brief specialty-aligned exposures can enhance engagement for some learners. Interestingly, Assimilating learners reported similarly high satisfaction regardless of specialty alignment, likely reflecting their preference for structured, conceptual learning rather than the specific clinical context.

Notably, lower satisfaction scores observed in hematology/oncology, psychiatry, and pediatrics may reflect small subgroup sizes or variability in instructional delivery rather than learning style effects alone. Prior simulation-based medical education research supports this interpretation, demonstrating that interactivity, distributed practice, feedback, and multi-modal instructional strategies exert a stronger influence on learner satisfaction than alignment with individual preferences [26]. Thus, while specialty-matched sessions may provide an incremental benefit, again, high-quality instructional design remains the most critical determinant of satisfaction across learners.

Practical significance and implications

While statistically significant differences between learning styles were observed, these were accompanied by generally small effect sizes, limiting their educational significance. Across all groups, students reported high satisfaction with procedural training, reinforcing its value as an effective and inclusive approach in pre-clerkship education.

Our findings extend the work of Newton et al., who underscored the persistence of learning style beliefs in medical education despite limited supporting evidence and growing concern over the "learning styles neuromyth" [9]. While much of that literature has focused on didactic settings, our study uniquely addresses experiential procedural training, a context theoretically suited for learning style differentiation, yet still demonstrates limited impact. However, we still found limited evidence that different learning styles require significant instructional variation. These findings further challenge the need for rigid learning style-matching frameworks in health professions education.

Similar themes have been reported by Ogut et al., who found that well-designed experiential learning modules in cross-sectional anatomy were highly valued across students with diverse interests and learning profiles [27]. Like our study, Ogut et al.'s findings highlight that while individual characteristics may influence preferences, instructional design quality, relevance, and engagement consistently emerge as the strongest predictors of positive educational outcomes.

That said, our results support the value of modest refinements. Incorporating brief pre-session demonstrations, reflective debriefs, or guided discussions may further optimize engagement, particularly for Diverging learners, without requiring substantial curricular overhaul. These adjustments complement the core drivers of instructional quality and may help maximize satisfaction across diverse learner profiles.

Ultimately, our findings reinforce that pedagogical quality, interactivity, relevance, and thoughtful instructional design remain the primary determinants of effective procedural education. Learning style-oriented adjustments may enhance experiences for some students but should complement, not replace, these foundational principles.

Limitations

Several limitations of this study warrant consideration. First, it was conducted at a single institution during a one-day event, limiting generalizability. Participation in the event was optional, introducing potential selection bias if students more interested or confident in procedural skills were more likely to attend. Additionally, students were able to self-select which specialty sessions to attend, which may have further introduced self-selection bias at the session level. Learners who opted into specific workshops may have had greater pre-existing interest or motivation related to the specialty or procedural content, potentially inflating satisfaction scores and limiting generalizability across a broader student population.

The peer-led, non-standardized nature of sessions introduced variability in instructor experience, interactivity, and resource availability, which may have influenced satisfaction independently of learning style. Prior research has demonstrated that variability in instructional design substantially impacts learner satisfaction regardless of individual learner characteristics [27].

Learning styles were self-reported using simplified Kolb-based descriptions rather than validated inventories, introducing potential classification bias. It should be noted that the self-categorization approach facilitated feasibility in a large cohort; however, the use of simplified learning style descriptions may have limited classification accuracy and does not fully replicate results that might be obtained using the validated Kolb Learning Style Inventory. Kolb's model itself remains debated, with concerns over its psychometric validity limiting the precision of learning style analyses [9].

Additionally, the study relied on self-reported satisfaction without the objective assessment of procedural competence or long-term clinical outcomes, limiting conclusions about the ultimate educational impact of the training.

From a statistical standpoint, analyzing individual survey items independently increased data points, potentially inflating statistical power and the risk of type I error. Although effect sizes were generally small, this approach remains a methodological limitation. Specialty-specific subgroup analyses were also limited by small sample sizes in certain fields (e.g., psychiatry, hematology/oncology), reducing confidence in those comparisons.

Finally, despite a relatively high 72% response rate, nonresponse bias cannot be excluded if nonrespondents systematically differed from survey participants. Collectively, these limitations suggest that while our findings offer valuable insights, caution is warranted in their interpretation. Future studies may also incorporate objective performance measures, such as Objective Structured Clinical Examination (OSCE)-based outcomes, to supplement subjective satisfaction data.

## Conclusions

Hands-on procedural training represents an effective and inclusive strategy in pre-clerkship medical education, with high satisfaction reported across diverse learning styles. While small differences by learning style emerged, these were of limited practical significance, suggesting that broadly applied, high-quality instructional design can meet the needs of most learners without necessitating rigid tailoring to individual preferences. Nevertheless, incorporating modest refinements, such as adding brief reflective components or specialty-aligned contextual framing, may further optimize engagement for select learner subgroups. Future research should examine how early procedural training influences clinical performance during clerkships and explore targeted instructional strategies that balance individualized support with scalable educational design.
